# Children’s Self-Regulation in Norway and the United States: The Role of Mother’s Education and Child Gender Across Cultural Contexts

**DOI:** 10.3389/fpsyg.2020.566208

**Published:** 2020-09-29

**Authors:** Ragnhild Lenes, Christopher R. Gonzales, Ingunn Størksen, Megan M. McClelland

**Affiliations:** ^1^Norwegian Centre for Learning Environment and Behavioural Research in Education, University of Stavanger, Stavanger, Norway; ^2^Center for Mind and Brain, University of California, Davis, Davis, CA, United States; ^3^Human Development and Family Sciences, Oregon State University, Corvallis, OR, United States

**Keywords:** cross-cultural, self-regulation, school readiness, measurement, maternal education level, gender

## Abstract

Self-regulation develops rapidly during the years before formal schooling, and it helps lay the foundation for children’s later social, academic, and educational outcomes. However, children’s self-regulation may be influenced by cultural contexts, sociodemographic factors, and characteristics of the child. The present study investigates whether children’s levels of self-regulation, as measured by the Head-Toes-Knees-Shoulders (HTKS) task, are the same in samples from Norway (*M*_age_ = 5.79; *N* = 243, 49.4% girls) and the United States (U.S.) (*M*_age_ = 5.65; *N* = 264, 50.8% girls) and whether the role of mother’s education level and child gender on children’s self-regulation differ across the two samples. Results showed that Norwegian and U.S. children had similar levels of self-regulation. Mother’s education level significantly predicted children’s self-regulation in the U.S. sample but not in the Norwegian sample, and this difference across samples was significant. Girls had a significantly higher level of self-regulation than boys in the Norwegian sample, but there were no gender differences in the U.S. sample. However, the effect of child gender on self-regulation did not differ significantly across the two samples. Results highlight the importance of cross-cultural studies of self-regulation.

## Introduction

In early childhood education and care (ECEC) contexts, children are socialized with peers through activities, such as social play, circle time, or waiting for a turn, which help them prepare for formal schooling. In these settings, children need to plan, cooperate, pay attention, inhibit impulses, and follow instructions. These behaviors depend on children’s self-regulation, which is the capability of controlling or directing one’s attention, thoughts, emotions, and actions (McClelland and Cameron, 2012). Self-regulation develops rapidly during the years before formal schooling (Center on the Developing Child at Harvard University, 2011), and children’s early self-regulation is critical for the transition to school and future academic achievement (Blair and Razza, 2007; Duncan et al., 2007; Welsh et al., 2010; von Suchodoletz et al., 2013; McClelland et al., 2014; ten Braak et al., 2018), as well as long-term health and educational outcomes (Moffitt et al., 2011; McClelland et al., 2013), income, and crime (Moffitt et al., 2011).

Most researchers suggest that children’s development consists of complex and bidirectional interactions between the child and the social context over time (e.g., Shonkoff and Phillips, 2000; Sameroff, 2009). The bioecological model of development (Bronfenbrenner, 1979; Bronfenbrenner and Morris, 2006) is one of the prevailing theoretical frameworks (Bornstein and Leventhal, 2015) that help provide a foundation for understanding these interactions. These interactions are influenced by individual differences in the development of children’s self-regulation, which can be explained by child characteristics (e.g., gender), socialization experiences, and sociodemographic factors (e.g., maternal education) (Eisenberg et al., 2014). Thus, for children growing up in different cultural contexts, such as Norway and the United States (U.S.), with different welfare systems, economic equality, availability of affordable ECEC, and a play-based vs. school readiness ECEC approach, the social experiences and the influence of maternal education and child gender may differ, which in turn may affect children’s development (Bronfenbrenner and Morris, 2006; Trommsdorff, 2009). Most prior cross-cultural studies of self-regulation have compared Western and Asian cultures (e.g., Oh and Lewis, 2008; Wanless et al., 2011a; Schirmbeck et al., 2020). The present study contributes by comparing self-regulation in Norway and the U.S. Both countries are characterized by high-income with Western individualistic cultures but offer different organization of the welfare state and different perspectives on the ECEC (ten Braak et al., 2019).

### Conceptual and Empirical Understandings of Self-Regulation

Different disciplines have taken a variety of approaches when investigating self-regulation and its related constructs (McClelland et al., 2014). Self-regulation is a multidimensional construct that broadly refers to the regulation of emotions, cognition, and behavior (McClelland et al., 2010). Moreover, self-regulation is understood to be composed of interrelated top-down and bottom-up components (Blair and Ursache, 2011; Blair and Raver, 2012). The bottom-up components are automatic, rapid, stimulus-driven reactivity and they do not require mental capacity, while the top-down components are related to executive functioning (EF) (Blair and Ursache, 2011; Blair and Raver, 2012; Nigg, 2017). EF is a high-level set of processes that include attentional or cognitive flexibility, working memory, and inhibitory control (Blair, 2002), and is often used and studied in cognitive disciplines (McClelland and Cameron, 2012). These higher-order cognitive processes are essential for goal-directed problem-solving in new situations and planning (Yeniad et al., 2013). EF is not synonymous with self-regulation; however, the EF components are cognitive processes that assist a child in broader aspects of self-regulation (Blair and Ursache, 2011). The Head-Toes-Knees-Shoulders (HTKS) task used in the present study has been found to be related to all three EF components in a behavioral self-regulation task (McClelland et al., 2014). Although EF processes have often been examined using materials and responses appropriate to the laboratory, the HTKS task measures the manifestation of those EF processes in real-world behavior (in an ecological setting) (McClelland and Cameron, 2012). This is consistent with the distinction of EF as a top-down cognitive process that enables self-regulation of a more automatic, bottom-up set of processes, such as the behavior a child would demonstrate in the HTKS task or in a social setting like a classroom.

### Development of Self-Regulation Across Cultures

The distinct role that culture plays in children’s development is of importance and aligns with the bioecological model of development (Bronfenbrenner and Morris, 2006). The bioecological model emphasizes the role of both proximal (micro-system factors) and distal (meso-, exo-, and macro-systems factors) systems of development. For example, the macro system in the bioecological model includes beliefs, values, and ideologies of the culture. Different beliefs, values, and ideologies may lead to different structural and socioeconomic organizations across cultures, such as the organization of the welfare and ECEC systems and the prevailing pedagogical approach. These differences across cultures may, in turn, affect the socialization practices (e.g., parents’ and teachers’ goals and expectations), the influence of sociodemographic factors (e.g., maternal education), and child characteristics (e.g., gender), and thus children’s development, including their self-regulation (Bronfenbrenner and Morris, 2006; Trommsdorff, 2009; Gestsdottir et al., 2014; McClelland et al., 2015). Country and culture are not synonymous, but for the current study, we refer to the participants’ shared nationality as their cultural context. However, we acknowledge that there is considerable cultural variation within a country as well (Minkov, 2013).

Children’s level of self-regulation may vary across socio-cultural orientations (e.g., child-rearing practices: independence and interdependence). For example, in cultures emphasizing an interdependent self (e.g., Asian collectivistic cultures), the goal of self-regulation may be tied on community ethics, including having harmonious relationships and the values of duty, respect, and obligation (Trommsdorff, 2009). For cultures emphasizing an independent self (e.g., Western cultures), the goal of self-regulation may be focused on autonomy and related independent identity (Trommsdorff, 2009). A recent review on self-regulation (EF) across cultures (nations) found that from preschool age through adolescence, East Asians outperformed Western counterparts on direct assessments of self-regulation (Schirmbeck et al., 2020). Less research, however, has examined and compared children’s self-regulation and the role of sociodemographic factors and child characteristics among children in cultures that focus on independence but differ in other important structural and philosophical ways. For example, Norway and the U.S., both Western cultures, are assumed to have more similar child-rearing practices but have different structural organizations and perspectives on ECEC and family policy.

Because differences in cultural contexts can also affect the way psychological assessments function (Oh and Lewis, 2008; Kline, 2016), is it important to establish that a measure of self-regulation (e.g., HTKS task; McClelland et al., 2014) possesses similar psychometric properties among 5-year-old children from Norway and the U.S. Thus, the present study first established measurement invariance across the two samples, which enabled a better comparison of whether mean levels of self-regulation and the influence of maternal education and child gender differed across cultural contexts (e.g., Kline, 2016).

### Early Childhood Contexts in Norway and the U.S.

Norway and the U.S. are high-income countries with a number of similarities. They are both individualistic cultures valuing independence, autonomy, human rights, and democracy. However, there are also several key differences that may influence the development of children’s self-regulation. For example, we know that economic equality and mobility are higher in Norway compared to the U.S. (Esping-Andersen, 2007; OECD, 2019). Wilkinson and Pickett (2009) have documented that countries with higher economic equality also have better mental and psychical health and higher academic outcomes. In 2013, only 6.8% of Norwegian children lived in poverty while the poverty rate was 21% in the U.S. (OECD, 2018), and poverty is known to be negatively related to self-regulation (Wanless et al., 2011b; Fitzpatrick et al., 2014; Blair and Raver, 2015). Moreover, Norway spends 3.3% of the gross national product on family benefits (child allowances, childcare support, income support during leave, and sole parent payments) while the U.S. spends 0.6% (OECD, 2017). For the purposes of the current study, we focused on differences in welfare systems and economic equality, availability of affordable ECEC, and a play-based approach prevalent in Norway that values unstructured play and social development, compared to a school readiness ECEC approach prevalent in the U.S. that includes a more structured approach to play and early academic achievement (OECD, 2006; Bennett, 2008).

#### The Cultural Context of Norway

Norway is a social-democratic country with a well-developed welfare system, including generous support for families, and a high priority on ECEC to promote social equality (Bambra, 2007; Norwegian Ministry of Education and Research, 2011, 2017; OECD, 2017). For example, parents have the right to share 12 months of paid parental leave after childbirth and adoption. Furthermore, the government highly subsidizes public and private ECEC, and families only pay 14% of annual ECEC expenditures (Lunder and Eika, 2017). Children aged 1–5 have the right to attend ECEC centers, and enrollment is very high. In 2012, 80.2% of the 1–2-year-olds were in ECEC centers, and 96.6% of the 3–5-year-olds (Statistics Norway, 2013). Most children (96%) go full time, which is up to 41 h a week.

Norwegian ECEC (public and private) is regulated by the *Framework Plan for the Content and Tasks of Kindergartens* (Norwegian Ministry of Education and Research, 2011, 2017). The framework plan reflects a play-based and child-centered approach (also called a social pedagogical or Nordic tradition), which emphasizes holistic learning based on children’s desire and curiosity for learning (OECD, 2006). The heart of this approach includes a focus on children’s current well-being and the intrinsic value of childhood. Early childhood is not merely a period in life that prepares children for education and adulthood (Tuastad et al., 2019). Free play and children’s autonomy are highly valued, and there is less emphasis on formal training for academic learning or self-regulation. The framework plan does not mention children’s need to develop self-regulation, and it contains no benchmarks for school readiness progress. Children spend considerable time in outdoor play in ECEC centers, 70% during the summer and 31% during the winter (Moser and Martinsen, 2010). A recent Norwegian study showed that children in ECEC centers spent 60% of the time on free play, and during free play, teachers were absent 45.5% of the time (Karlsen and Lekhal, 2019). The ECEC centers are usually organized in groups of nine children aged 1–2-years and groups of 18 children for the 3–5-year-olds. The groups’ main staff is one teacher with a bachelor’s degree and two assistants. Children attend the same ECEC center until they start first grade of formal schooling at the end of August the year they turn 6 (the cut-off date is January 1st).

Characteristics of the Norwegian society, such as the well-developed welfare system with a strong family service orientation, social and economic equality, and availability of affordable ECEC, as well as a play-based and child-centered approach, may promote opportunities for the Norwegian children to develop self-regulation (Esping-Andersen, 2007; Wilkinson and Pickett, 2009). According to Esping-Andersen (2007), high-quality ECEC is one way to help ensure that all children receive a strong foundation prior to school. Moreover, researchers argue that free play (especially social pretend play) and the autonomy that is common in the play-based approach are important for the development of self-regulation (Vygotsky, 1978; Diamond et al., 2007; Center on the Developing Child at Harvard University, 2011; Diamond and Lee, 2011; Engel et al., 2015). For example, during pretend play, children must remember their own and other’s roles, inhibit acting out of the character and flexibly adjust to their playmates’ improvisations (Diamond and Lee, 2011). Thus, these activities challenge and promote EF processes and self-regulation abilities.

#### The Cultural Context of the U.S.

The U.S. is a democratic country, where the state or federal provision of welfare is minimal (Bambra, 2007). The country has a liberal market economy, which approaches the daycare (especially under three years) as a private responsibility for parents and not a public responsibility (Bennett, 2008). The use of care and education depends on the age of children, employment status of parents, household income, and access to free or subsidized care (Early Care and Education Profiles, 2018). The country has a two-tier organization of the services: child care for children from 0 to 3 years, followed by a pre-primary education for the 3–5-year-olds (Bennett, 2008). ECEC institutions differ greatly in their requirements, operational procedures, regulatory frameworks, staff-training, and qualifications. ECEC is expensive for families, and they have to fund as much as 72% of annual childcare expenditures (in states where the U.S. data were collected; Early Care and Education Profiles, 2018). However, there are some programs providing support to low-income families. Head Start is an example of a free federal preschool program for children aged 3–5 years. According to the Early Care and Education Profiles (2018) report, only 18% of the children under age 3 attended daycare, and 46% of the children aged 3-and-4 years were enrolled in preschool in 2016.

Overall, there are large variations in the experiences that young children receive in the U.S., although many ECEC programs have a school readiness approach, which focuses on teaching cognitive and pre-academic skills (OECD, 2006; Bennett, 2008). Moreover, compared to the Norwegian ECEC system, they spend less time on free play (30%; Chien et al., 2010). Children’s self-regulation may be more systematically supported in a school readiness approach compared to a play-based approach. This may be because of an intentional focus on activities that promote self-regulation, such as having to pay attention to and remember instructions and demonstrate self-control (Gestsdottir et al., 2014). In addition, in the U.S., most children start formal schooling in kindergarten when they are 5 years old (the cut-off date for children in the current study was September 1st), which has a stronger focus on school readiness and academic learning, whereas in Norway children do not enter formal schooling until they are 6 years old. Based on the pedagogical approach in the ECEC context and the earlier transition to formal schooling in the U. S. compared to Norway, there may be greater opportunities to practice self-regulation in the U.S. compared to Norway. This may be especially true for children who are low in self-regulation and who may benefit from structured activities prior to school entry (Zambrana et al., 2020). Thus, it may be that each culture has different characteristics that help promote self-regulation.

### Predictors of Children’s Self-Regulation

According to the bioecological model of development, children’s cultural contexts also influence the role of children’s socioeconomic background and gender in children’s socialization processes (Bronfenbrenner and Morris, 2006). Prior research has found that children’s self-regulation is related to maternal education and child gender (Kishiyama et al., 2009; Matthews et al., 2009; Sektnan et al., 2010; Wanless et al., 2011b, 2013; DiPrete and Jennings, 2012; Størksen et al., 2015; Backer-Grøndahl and Nærde, 2017). However, it is unclear whether the influence of these factors on children’s self-regulation differs across cultural contexts.

#### Socioeconomic Background

Socioeconomic background affects children’s socialization, which leads to variations in their social, emotional, cognitive, and physical functioning (Conger and Donnellan, 2007). Parental socioeconomic status (SES) is indicated by income, education, and occupation (Conger and Donnellan, 2007). In particular, maternal education has been a good indicator of SES in studies of child development (Bornstein et al., 2003; Hoff et al., 2012). For example, parents with higher education levels may place a stronger priority on activities, goods, and services that foster academic and social competence, compared to parents with lower education levels (Conger and Donnellan, 2007; Conger and Dogan, 2014). Research has indicated that children in poorer home environments, as measured by the home literacy environment, have significantly lower self-regulation than their peers (McClelland et al., 2000). Prior research has also found that parent’s stimulation mediates the relationship between parental education and child competence (Bradley and Corwyn, 2003). Thus, the relation between maternal education and children’s self-regulation may reflect the number of opportunities (e.g., in everyday interactions and pre-academic-, music-, and outdoor-activities) children receive to practice their self-regulation.

Prior research conducted in the U.S. has reported that children’s socioeconomic background predicts their self-regulatory skills (Sektnan et al., 2010; Wanless et al., 2011b; Conway et al., 2018). One study with samples from the U.S. investigated the effect of maternal education on children’s self-regulation trajectories (using the HTKS task) and found that early developers generally had mothers with higher education levels (Montroy et al., 2016). Another study with samples from France, Iceland, and Germany found that maternal education did not predict children’s self-regulation in any samples, using the HTKS task (Gestsdottir et al., 2014). Even though Norway has relatively little poverty and economic and social equality is high, the socioeconomic background is an important predictor of school achievement (Bakken and Elstad, 2012). Moreover, two prior Norwegian studies have found some evidence for associations between socioeconomic background and children’s self-regulation. Backer-Grøndahl and Nærde (2017) found that socioeconomic background (parent education level and whether families live in poorer housing) predicted cool (cognitive aspects of self-regulation) but not hot (emotional aspects of self-regulation) self-regulation. In contrast, Størksen et al. (2015) documented that socioeconomic background (parent’s education level and income) predicted teacher reported self-regulation in children, but only predicted directly assessed self-regulation (e.g., HTKS task) for girls and not boys. Although socioeconomic background has predicted children’s self-regulation in Norway and the U.S, research has not examined if this relationship is significantly different across the cultures.

#### Child Gender

Research in Norway and the U.S. has demonstrated that girls tend to have higher self-regulation than boys in preschool and kindergarten (Matthews et al., 2009; DiPrete and Jennings, 2012; Størksen et al., 2015; Backer-Grøndahl and Nærde, 2017), although some findings from the U.S. using the HTKS task are inconsistent (McClelland et al., 2007; Schmitt et al., 2014). Moreover, some differences have been detected in research across various cultures. One study showed gender differences in self-regulation, as measured by the HTKS task, in the U.S. sample, but no significant gender differences the samples from the Asian cultures (Taiwan, China, or South Korea) (Wanless et al., 2013). Another European study also using the HTKS task found that girls scored higher than boys on self-regulation in an Icelandic sample but this was not found in the French and German samples (Gestsdottir et al., 2014). However, a German study found that although 4-year-old girls showed higher self-regulation on the HTKS task, boys caught up the following 2 years (Gunzenhauser and von Suchodoletz, 2015). In line with these results, a U.S. study found that girls were associated with earlier development trajectories of self-regulation while there were more boys in the later developers’ group (Montroy et al., 2016). Finally, a recent review investigating similarities and distinctions across countries in the development of self-regulation and EF found that girls performed better than boys on direct assessment and teacher and parent ratings in both Western and East Asian samples (Schirmbeck et al., 2020).

Many gender theories acknowledge that a combination of biological and social factors influence gender development (Leaper and Friedman, 2007; Reilly et al., 2018). The influence of culture on gender differences may be seen in different expectations for self-regulatory behavior among boys and girls across cultures, and through different socialization processes (Gestsdottir et al., 2014). Norway and the U.S. are Western cultures that emphasize gender equality. In spite of this, in both countries, there is evidence that girls and boys experience different expectations based on traditional gender patterns (Chick et al., 2002; Meland and Kaltvedt, 2017). One Norwegian study found that girls were praised for characteristics, such as being caring, helpful, responsible, and conscientious, while the staff affirmed boys’ strength and physical characteristics (Meland and Kaltvedt, 2017). Teachers expected girls to sit still, wait for help, and play quietly, while the boys were allowed to be noisy, climb, and jump. A study conducted in a U.S. preschool found similar differences in staff expectations for girls and boys (Chick et al., 2002), as found in the Norwegian study. Thus, ECEC staff in both countries may expect girls to behave in a more self-regulated manner compared to boys (Chick et al., 2002; Matthews et al., 2009; Størksen et al., 2015; Meland and Kaltvedt, 2017).

### Measuring Self-Regulation Across Cultural Contexts

In order to have a valid group comparison, it is important to establish that the measurement functions similarly across groups. This is more generally referred to as measurement invariance (van de Schoot et al., 2012; Kline, 2016). There are four levels of measurement invariance, which get more restrictive for each level and help establish how similar the measurement functions in each group (Kline, 2016). The least restrictive level, configural invariance, establishes that the measure consists of the same general underlying structure *via* confirmatory factor analysis (CFA). In the next levels, factor loadings (weak invariance), intercepts (strong invariance), and finally, residual variances (strict invariance) in the CFA are constrained to be equal across groups (see “Analytic Strategy” section for further descriptions). Strong measure invariance is required in order to have meaningful interpretations when comparing differences between groups (van de Schoot et al., 2012; Kline, 2016). When strong invariance is established, it means that if two children from two different groups have the same underlying levels of self-regulation, they are also more likely to obtain the same score on the measure (Kline, 2016). In addition to measurement invariance, research suggests that when nationally representative samples are not possible, having matched samples that are as similar as possible help ensure a valid group comparison in cross-cultural studies (Minkov, 2013). This helps ensure that differences found across the samples are not due to sample-specific characteristics.

### The Present Study

The main goals of this study were to investigate (1) children’s level of self-regulation (using the HTKS task) across a Norwegian and a U.S. sample and (2) the influence of mother’s education level and child gender on children’s self-regulation across the two samples.

Prior studies have reported that the HTKS task has shown strong psychometric properties across cultural contexts (Wanless et al., 2011a; Gestsdottir et al., 2014; McClelland et al., 2014; Størksen et al., 2015). However, strong measurement invariance is required to compare group means (van de Schoot et al., 2012; Kline, 2016), so we first examined measurement invariance for the HTKS task across the two samples.

No prior studies have directly compared Norwegian and U.S. children’s self-regulation. We expected that both cultures had characteristics that would promote children’s self-regulation in different ways. For example, Norway emphasizes free play in ECEC, which for some children, can be beneficial in developing self-regulation. Moreover, there is low child poverty and economic inequality, a well-developed social democratic welfare system, a strong family service orientation, and earlier and higher attendance to ECEC in Norway compared to the U.S., all of which can promote Norwegian children’s self-regulation (OECD, 2016, 2019; Lunder and Eika, 2017; Early Care and Education Profiles, 2018). In the U.S., there is some evidence that children have opportunities to practice self-regulation because of the predominant school readiness approach in ECEC and kindergarten, compared to children in unstructured play-based ECECs in Norway (Gestsdottir et al., 2014). Thus, we did not expect significant differences in self-regulation across the cultures.

There is some evidence to expect maternal education to significantly predict children’s self-regulation in both cultures (e.g., Sektnan et al., 2010; Backer-Grøndahl and Nærde, 2017). However, due to the sociopolitical differences across the two cultures, we expected maternal education to be a significantly stronger predictor for the U.S. children’s self-regulation than for the Norwegian. Finally, based on prior evidence (Chick et al., 2002; Matthews et al., 2009; Størksen et al., 2015; Meland and Kaltvedt, 2017), we expected girls to score higher on the self-regulation measure compared to boys in both societies and the influence of gender on self-regulation to be equal across the two samples.

## Materials and Methods

### Participants

In the present study, we used samples from research projects in Norway (243 children) and the U.S. (264 children). To get the samples as similar as possible in age, we used data from the spring of the last year of ECEC in the Norwegian sample and from the fall of kindergarten in the U.S. sample. The mean age in the Norwegian sample was 5.79 years (*SD* = 0.29), and the mean age in the U.S. sample was 5.65 years (*SD* = 0.31). Thus, the samples on average differed only about one and a half months in mean age.

#### Norway

Data from the Norwegian sample derived from the Skoleklar [School readiness] research project. The sample of children and families were from a primarily rural county in Norway. All children (*N* = 287) who were in their last year of ECEC in 2011 in a municipality in the Norwegian west coast were invited to participate, using a convenience sampling approach. A total of 243 children (84.7%) had parental consent to participate. Among these, there were 119 girls (49.4%) and 124 boys (50.6%). Data used in the present study derived from 19 centers and were collected in spring 2012, the last year children attended ECEC. The median age of starting in ECEC was 18 months. For more details of this sample, see previous descriptions in Størksen et al. (2015).

The sample had no group assessed in another language than Norwegian, but 13 children (5.3%) had an immigrant background where both parents were born in another country than Norway (11 different countries). These children had a mean sum score of 45.58 (*SD* = 24.74) on the HTKS task, which was not significantly different from the scores of children that had both or one parent born in Norway (*M* = 52.51, *SD* = 20.04).

#### The United States

Data from the U.S. sample derived from children recruited from 17 local preschools in a rural area in the Pacific Northwest as part of a larger study (Touch your toes! Devleoping a new Measure of Behavioral Regulation), examining children’s self-regulation in the transition to kindergarten. The principal investigator contacted preschool directors *via* telephone, e-mail, and individual meetings to invite them to be a part of the study using a convenience sampling approach (i.e., preschools that were accessible and willing to participate in the study). For more details of this sample, see previous descriptions in McClelland et al. (2014) and Schmitt et al. (2017). The data used in the present study were collected in the fall of kindergarten (2012) and included 310 children attending 38 schools.

At fall in kindergarten, 46 children (15%) were identified as English language learners (ELL) and were assessed in Spanish. Preliminary analyses showed that these children had significantly lower scores on the HTKS task compared to children tested in English (*M* = 28.80, *SD* = 28.14, and *M* = 53.24, *SD* = 21.58 respectively). To ensure a more valid comparison and because the Norwegian and the U.S. samples were convenience samples, rather than nationally representative samples, samples were matched on key variables of interest (Minkov, 2013). In other words, to ensure that self-regulation differences were not due to characteristics of the subgroup of children assessed in Spanish (ELL) in the U.S. sample (Banks et al., 2006), we excluded these 46 children, which left a total U.S. sample size of 264 children. Among the 264 children, 111 children (42%) were enrolled in Head Start. The sample included 49.2% of boys and 50.8% of girls. The median of months in daycare (0–3) among the 264 children was 5 months, and 90 children had no daycare experience. Furthermore, the median of months in preschool was 12 months.

#### Demographic Information

Parents completed demographic surveys in both samples. An education level of a high school diploma or less was scored as zero (NO = 42.9%, U.S. = 31.3%). Some college or an associate’s degree was scored as one (NO = 8.6%, U.S. = 13.5%). A bachelor college degree (BA, BS, etc.) was scored as a two (NO = 22.9%, U.S. = 26.9%), and advanced degree (MA, MS, MD, Ph.D., etc.) was scored as a three (NO = 25.4%, U.S. = 28.4%). Mother’s median education level in the Norwegian sample was some college or an associate’s degree (*M* = 1.31, *SD* = 1.26). In the U.S. sample, the mother’s median education level was a bachelor’s college degree (*M* = 1.53, *SD* = 1.20) when ELL children were excluded. Overall, the U.S. sample had a higher maternal education than the Norwegian sample with (*M* = 1.41, *SD* = 1.22) or without ELL children included.

In the Norwegian sample, parents reported their minority status by indicating their country of birth. Parents reported being born in 21 different countries in addition to Norway. If one of the parents (5.8%) or both (5.3%) were born in another country than Norway (or Scandinavia), children were scored as minority status (11.1%). In the U.S. sample, parents reported their child as White (69.7%), African American (0.4%), Latino/Hispanic (4.9%) Asian/Pacific Islander (3.4%), Middle Eastern (0.8%), more than one race or ethnicity (14.4%) or other (0.8%). All categories, except White, were scored as minority status (24.6%).

Mothers with minority status in the Norwegian sample had a median education level of some college or an associate’s degree (*M* = 1.15, *SD* = 1.29), while the median education level of those not being a minority was between some college or an associate’s degree and a bachelor college degree (*M* = 1.32, *SD* = 1.26). In the U.S. sample, mothers with minority status had a median education level of some college or an associate’s degree (*M* = 1.30, *SD* = 1.23), and mothers not having minority status had a median educational level of a bachelor college degree (*M* = 1.59, *SD* = 1.19).

### Missing Data

The Norwegian sample had 0.8% missing on the HTKS task, and the U.S. sample had 1.5–1.9% missing on the HTKS. The Norwegian sample had 2.9% missing on the minority status variable, while the U.S. sample had 5.7%.

Maternal education had 21.2% (*N* = 56 cases) missing in the U.S. sample. We conducted *t*-tests and found that there were significantly more children with minority status that were missing on maternal education. Moreover, those with missing data on maternal education had significantly lower mean sum scores on the HTKS task compared to those that had reported on this variable (respectively: *M* = 45.48, *SD* = 23.07, and *M* = 55.22, *SD* = 20.78; see below for methods of dealing with missing data). The Norwegian sample had only 1.3% (three cases) missing on maternal education, and there were no significant differences between those with and without data on the variable.

### Procedure

#### Norway

A test battery of school readiness assessments was administered individually with the use of computer tablets. In addition to the HTKS task, the battery consisted of one additional self-regulation measure (teacher report) and academic measures (vocabulary, math, and phonological awareness). Results from these other tasks are outside the scope of the current study but are reported elsewhere (Lens et al., 2020). Children were tested in Norwegian in a one on one session with a research assistant in an adjacent room in their ECEC center to reduce any excess distraction during testing. Children completed the test battery in one test session, and it took 30–40 min. The parents reported their education level and country of birth, date of the child’s birth, and gender on a questionnaire. The questionnaires were organized by the ECEC centers in collaboration with the project administrators.

#### The United States

Children were assessed individually on a battery of school readiness assessments in their schools in a one on one session with a research assistant. In addition to the HTKS task, the battery consisted of other self-regulation measures (the Day-Night and DCCS) and academic measures (the Woodcock-Johnson tests). Descriptive results from these measures are reported elsewhere (McClelland et al., 2014; Schmitt et al., 2017). Children completed the battery of assessments over two to three 15-min sessions within 2 weeks. All sessions were conducted in a quiet corner or an adjacent room or hallway to the classroom. Parents were sent demographic questionnaires *via* the mail and were asked to return them by the completion of the study.

### Measures

#### Self-Regulation

Self-regulation in both samples was assessed with the HTKS (McClelland et al., 2014). The test is a short game appropriate for children aged 4–8 years and includes three parts. Each of the three parts has one practice section (four items) and one following test section (10 items). In the present study, we incorporated both the practice sections and the test sections. There are a total of 12 practice items and 30 test items with scores of 2 points for a correct response, 1 point for a self-correct response, and 0 for an incorrect response. For each of the three parts, children do not move onto the next part of the test if they do not receive at least four (out of twenty) points on the test section.

In the first part of the HTKS task, children are asked to touch the opposite body part of what is presented to the child. In the second part, two additional body parts are added, and in the third part, the rules are switched. The HTKS task requires children to integrate several executive function skills, namely (1) paying attention to the instructions, (2) using working memory to remember and execute new rules, (3) using inhibitory control through inhibiting the natural response to the instructor’s command, and (4) use cognitive flexibility and working memory when rules are switched (Cameron Ponitz et al., 2009a; McClelland et al., 2014).

In the Norwegian sample, the item level data of the HTKS task were not available. We, therefore, only had sum scores for the practice and test sections of the measure and could not calculate the Cronbach’s alpha reliability. In the U.S. data, where the item-level data were available, the reliability was *α* = 0.96 (42 items). The HTKS task has shown good psychometric properties in previous studies conducted in the U.S., Asia, and Europe (Cameron Ponitz et al., 2009a; von Suchodoletz et al., 2013; Wanless et al., 2013), with Cronbach’s alpha reliability ranging from 0.92 to 0.94 (McClelland et al., 2014). In data from a recent Norwegian research project (Rege et al., 2019), with a similar age group, the HTKS task showed a Cronbach’s alpha of *𝛼* = 0.87 (30 test items).

### Analytic Strategy

Because children were nested in different ECEC centers and schools in the two samples, we calculated intra-class coefficients (ICC; the proportion of the total variability in the outcome that is attributable to the classes; Geiser, 2013). The average cluster size was 11.48 in the Norwegian sample, and ICCs ranged between 0.001 and 0.046 for all the HTKS practice and test sections, and it was 0.034 for the sum score of the HTKS task. In the U.S. sample, the average cluster size was 6.68 and ICCs ranged between 0.018 and 0.079 for all the HTKS practice and test sections, and it was 0.063 for the sum score of the HTKS task. As the ICCs were not substantial in the two samples (Hox, 2002), analyses adjusting for potential nested effects were not conducted.

Maternal education in the U.S. sample had 21.2% missing. As missing on this variable was predicted by the minority status variable, we included minority status as a covariate in the further analyses. Furthermore, to appropriately deal with missingness, we used full information maximum likelihood estimators (FIML), which can provide more optimal solutions compared to traditional missing data handling techniques (Enders, 2010).

To test the measurement invariance of the HTKS task across the two samples, we conducted a series of CFAs using Mplus version 7.3 (Muthén and Muthén, 1998–2015). Children’s sum scores for the practice and test items subsections in the three parts of the HTKS task were used as individual indicators in the CFAs; thus, there were six indicators. We proceeded in a stepwise fashion from the least restrictive model (configural invariance) to the most restrictive model (strict invariance; van de Schoot et al., 2012; Kline, 2016). Configural invariance was tested by constraining the latent structure to be equal across the Norwegian and the U.S. samples. Factor means were fixed to 0, and factor variances were fixed to 1. Weak invariance was tested by also equating the unstandardized factor, strong invariance by equating unstandardized intercepts, and, finally, strict invariance by equating unstandardized residual errors.

For each step in the analyses, the model fit was assessed using the Chi-square statistics, the comparative fit index (CFI and TLI; a value greater than 0.95), the root-mean-square error of approximation (RMSEA; a value less than 0.05) and the root-mean-square residuals (SRMR; a value less than 0.10; Hox and Bechger, 1999; Kline, 2016). Because the models we tested were nested, Chi-square difference tests were used to compare the models. We used the Satorra Bentler correction due to the MLR estimator used (Satorra and Bentler, 2010; Muthén and Muthén, 2018). In the case of no significant Chi-square test, the more restrictive model was favored. We also tested that the more restricted model did not decrease more than 0.01 in the CFI value compared to the less restricted model (Cheung and Rensvold, 2002).

If strong measurement invariance is established, we can compare group scores on the latent variable (van de Schoot et al., 2012; Kline, 2016). To investigate whether children’s levels of self-regulation was significantly different across the samples, we tested if latent factor means and the correlation between them differed significantly across Norwegian and U.S. children. Factor means and variances were allowed to vary freely, and the first factor loading for each of the factors was fixed to one. The U.S. sample was the reference group, and the factor means in the Norwegian sample were compared to the U.S. means. The correlation between the two factors was compared between the samples using a Wald test.

We examined whether maternal education and child gender predicted children’s self-regulation differently across the two samples by contrasting two models (structural equation modeling; SEM). In the first model, maternal education was allowed to vary freely in predicting the HTKS factors across the samples. In the second model, the parameters were constrained to be equal across the samples. The model with constrained paths across the samples was compared to the model where maternal education was free by computing a Chi-square difference test (Satorra Bentler correction). We repeated this procedure for gender as a predictor of the HTKS factors. Child age and minority status were used as covariates.

## Results

### Descriptive Statistics

To check for potential bias due to outliers (van de Schoot et al., 2012), data were screened by looking at histograms and boxplots in SPSS, and by checking in Mplus if any cases had a Cook’s distance greater than 1 (Cohen et al., 2003; Field, 2013) or if the value of Mahalanobis distance was *p* < 0.001 (Tabachnick and Fidell, 2014). Cases that had values indicating they were outliers were investigated further. We also did the analyses with outliers excluded. However, the results did not differ; thus, outliers were included in further analyses.

Descriptive statistics for the two samples are reported in Table 1. For both samples, the mean performance on the three test sections of the HTSK decreased between part one and part three. In the Norwegian sample, 87.7% of the children advanced to test part two, while 86.4% of the children in the U.S. sample advanced. Furthermore, 71.6% of the Norwegian children and 67.4% U.S. children advanced to test part three.

**Table 1:** Descriptive for all study variables.

	NO M(*SD*)	US M(*SD*)	*ES*	NO skewness/ kurtosis	US skewness/ kurtosis	NO% floor/ ceiling	US% floor/ ceiling
Age T1 NO = 242, US = 264	5.79 (0.29)	5.65 (0.30)	0.47^***^				
Percent male NO = 241, US = 264	50.6	49.2	0.03 ns^a^				
Percent minority NO = 237, US = 249	11.4	26.1	0.39^***^^,^ ^a^				
Mother’s education NO = 240, US = 208	1.31 (1.26) Median = 1	1.52 (1.20) Median = 2	−0.17 ns^a^	0.17/−1.64	−0.11/−1.54	42.9/25.4	31.3/28.4
HTKS P1 NO = 241, US = 260	6.91 (1.71)	6.90 (2.10)	0.01 ns	−2.39/6.33	−2.32/ 4.54	2.5/52.3	5.4/62.7
HTKS T1 NO = 241, US = 260	15.14 (5.61)	15.57 (5.78)	−0.08 ns	−1.47/1.14	−1.70/1.69	5.4/15.4	6.2/18.8
HTKS P2 NO = 241, US = 260	6.47 (2.43)	6.39 (2.44)	0.03 ns	−1.79/1.99	−1.82/2.08	8.7/53.1	10.0/45.4
HTKS T2 NO = 241, US = 260	11.78 (6.38)	12.52 (6.26)	−0.12 ns	−0.62/−0.94	−0.90/−0.42	11.6/5.0	12.3/5.8
HTKS P3 NO = 241, US = 259	4.38 (2.77)	4.26 (2.82)	0.04 ns	−0.42/−1.09	−0.27/−1.23	20.7/15.8	20.5/17.4
HTKS T3 NO = 241, US = 259	7.54 (6.99)	7.58 (7.03)	−0.01 ns	0.41/−1.30	0.34/−1.40	27.8/5.0	31.3/4.6
HTKS total score NO = 241, US = 259	52.22 (20.18)	53.24 (21.61)	−0.05 ns	−0.93/0.28	−1.08/0.51	1.7/0.0	4.2/0.4

NO sample, Norwegian sample; US sample, United States sample; HTKS, Head-Toes-Knees-Shoulders task; P1, sum of practice items part 1; T1, sum of test items part 1; P2, sum of practice items part 2; T2, sum of test items part 2; P3, sum practice items part 3; T3, sum of test items part 3, and HTKS total score, sum of all practice and test items; ES, Cohen’s D.

a Pearson’s Chi-square test.

*** *p* < 0.001.

Although we had not yet established measurement invariance, we tested mean-level differences between the Norwegian and U.S. samples on the subsections of the HTKS task in the preliminary analyses by conducting independent samples *t*-tests. Results showed that none of the practice or test sections in the HTKS task differed significantly between the groups (see Table 1).

Moreover, there was no significant difference in maternal education, *χ*(3) = 7.390, *p* = 0.060 between the two samples, but there was a significant difference in the proportion of minority status between the groups, *χ*(1) = 17.126, *p* < 0.001. However, independent sample t-tests showed no significant differences in scores in the HTKS subsections between children having minority status or not in either of the samples. Moreover, maternal education did not significantly differ between being minority status or not, in the U.S. sample χ(3) = 2.464, *p* = 0.483, or the Norwegian sample *χ*(3) = 7.019, *p* = 0.071. The small mean age difference (*M* = 1.8 months) between the two samples was statistically significant, *t*(504) = −5.123, *p* < 0.000.

As shown in Table 2, gender correlated significantly with five of six of the HTKS subsections in the Norwegian sample. In contrast, gender did not correlate significantly with any of the HTKS subsections in the U.S. sample. In the U.S. sample, maternal education correlated significantly to all HTKS subsections. However, this was not the case with the Norwegian sample, where there were no significant correlations between the HTKS subsections and maternal education. Minority status did not correlate significantly with any of the HTKS subsections in the two samples.

**Table 2:** Correlations for all study variables. The Norwegian sample above the diagonal in the top panel and the U.S. sample below.

	1.	2.	3.	4.	5.	6.	7.	8.	9.	10.	11.
1. Child age	-	−0.04	0.04	0.00	0.04	0.13^*^	0.10	0.14^*^	0.10	0.09	0.14^*^
2. Child gender	0.09	-	−0.07	0.04	−0.11	−0.14^*^	−0.29^***^	−0.16^*^	−0.20^**^	−0.26^***^	−0.25^***^
3. Mother’s education	−0.04	−0.02	-	−0.04	0.10	0.10	0.01	0.11	0.11	0.11	0.12
4. Minority	−0.10	−0.09	−0.10	-	−0.09	−0.05	−0.00	−0.12	−0.07	0.00	−0.07
5. HTKS P1	0.05	−0.09	0.27^***^	−0.09	-	0.47^***^	0.42^***^	0.36^***^	0.35^***^	0.18^**^	0.49^***^
6. HTKS T1	0.09	−0.02	0.25^***^	−0.07	0.64^***^	-	0.66^***^	0.63^***^	0.47^***^	0.39^***^	0.80^***^
7. HTKS P2	0.04	−0.04	0.24^***^	−0.05	0.68^***^	0.77^***^	-	0.62^***^	0.51^***^	0.41^***^	0.75^***^
8. HTKS T2	0.14^*^	−0.03	0.24^***^	−0.05	0.58^***^	0.72^***^	0.69^***^	-	0.63^***^	0.55^***^	0.87^***^
9. HTKS P3	0.07	−0.06	0.24^**^	0.03	0.49^***^	0.59^***^	0.55^***^	0.70^***^	-	0.57^***^	0.76^***^
10. HTKS T3	0.14^*^	−0.04	0.17^*^	−0.07	0.38^***^	0.44^***^	0.39^***^	0.55^***^	0.65^***^	-	0.77^***^
11. HTKS total score	0.13^*^	−0.05	0.28^***^	−0.06	0.70^***^	0.85^***^	0.79^***^	0.89^***^	0.81^***^	0.77^***^	-

HTKS, Head-Toes-Knees-Shoulders task; P1, sum of practice items part 1; T1, sum of test items part 1; P2, sum of practice items part 2; T2, sum of test items part 2; P3, sum practice items part 3; T3, sum of test items part 3, and HTKS total score, sum of all practice and test items.

* *p* < 0.05;

** *p* < 0.01;

*** *p* < 0.001.

#### Establishing Measurement Invariance

**Figure 1:**
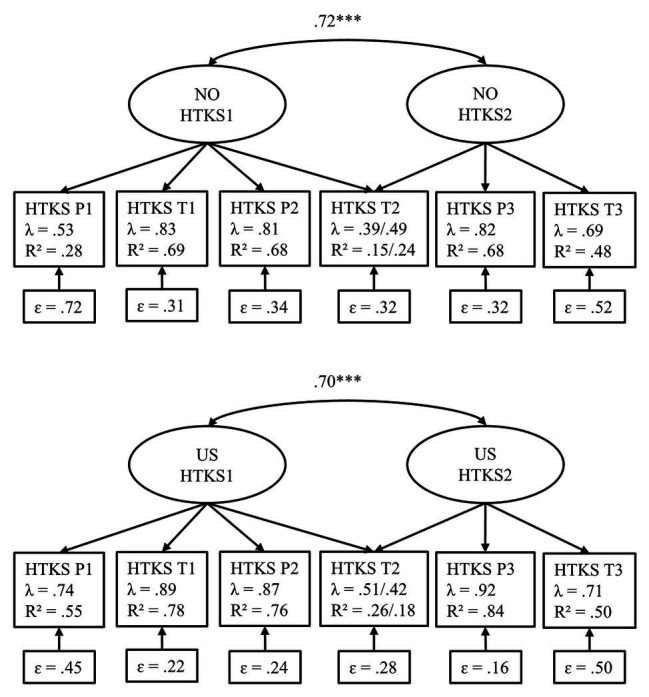
Standardized parameter estimates for self-regulation (HTKS1 and HTKS2) in Norwegian and US samples using configural model. Factor variances were fixed to 1, and factor means fixed to 0. All factor loadings estimated freely for each sample. NO sample, Norwegian sample; US sample, United States sample; HTKS, Head-Toes-Knees-Shoulders task; P1, sum of practice items part 1; T1, sum of test items part 1; P2, sum of practice items part 2; T2, sum of test items part 2; P3, sum practice items part 3; T3, sum of test items part 3. ^***^p < 0.001.

When investigating the initial factor structure of the HTKS task, preliminary analyses indicated that a one-factor solution did not adequately fit the data [*χ*^2^ (18) = 154.08, *p* < 0.001, RMSEA = 0.173, CFI = 0.913, TLI = 0.855, SRMR = 0.055]. We continued by investigating a two-factor solution that prior research has also supported. As children get older, the HTKS task shows a greater differentiation because children also manage to advance to the harder sections of the task, which places additional demands on children’s cognitive flexibility and working memory (McClelland et al., 2014). As a result, easier, early parts of the HTKS task, which primarily taps children’s inhibitory control, tended to load significantly onto the first latent factor (HTKS1), whereas the harder, later parts of the measure, which have additional requirements on children’s cognitive flexibility and working memory, loaded significantly onto the second latent factor (HTKS2). Results from the CFA analysis showed that the same underlying two-factor structure was valid for the Norwegian and U.S. samples and showed good overall model fit (see Table 3, Model 1; Configural invariance). Thus, we utilized the two-factor (HTKS1 and HTKS2) model for all subsequent analyses. Figure 1 shows that all factor loadings for all indicators of the HTKS factors were statistically significant. As shown in Table 3, the more strict models did not have statistically significantly worse fit to the data (see *χ^2^diff*), and CFI did not decrease more than 0.01 when comparing them to the less strict models (e.g., weak vs. configural and strong vs. weak). Thus, measurement invariance was established, and we could have a valid comparison of the two samples in further analyses.

**Table 3:** Test of measurement invariance for a two-factor solution of the Head-Toes-Knees-Shoulders task across the Norwegian and U.S. samples.

	*χ*^2^	*p*	*df*	Model comparison	χ^2^diff^*^	Δ*df*	RMSEA	SRMR	CFI	TLI
Single group Solution										
NO (*n* = 241)	13.538		7				0.062	0.023	0.989	0.977
US (*n* = 262)	6.559		7				0.000	0.012	1.000	1.001
Model 1. Configural	19.295	0.154	14				0.039	0.018	0.996	0.992
Model 2. Weak^a^	33.796	0.038	21	2 vs. 1	13.219, *p* = 0.067	7	0.049	0.100	0.991	0.987
Model 3. Strong^a^^,^ ^b^	42.238	0.031	27	3 vs. 2	8.388, *p* = 0.211	6	0.047	0.100	0.989	0.988
Model 4. Strict^a^^,^ ^b^^,^ ^c^	55.030	0.009	33	4 vs. 3	12.052, *p* = 0.061	6	0.052	0.099	0.984	0.986

NO sample, Norwegian sample; US sample, United States sample.

a All factor loadings are equal across samples.

b All intercepts are equal across samples.

c All residuals are equal across samples.

* Satorra Bentler Correction for chi-square difference tests was used when comparing the models due to the MLR estimator used.

### Children’s Levels of Self-Regulation Across a Norwegian and a U.S. Sample

In the Norwegian sample, the mean of the HTKS1 factor was 0.064 lower, (*p* = 0.600) compared to the mean in the U.S. sample, and the mean of the HTKS2 factor was 0.015 higher (*p* = 0.957; using the strong invariance model; see Figure 1 for an overview over subsections included in the HTKS1 and HTKS2 latent factors). Thus, we found no significant differences between the sample means on either of the HTKS1 and HTKS2 latent factors. A Wald test [0.783(1), *p* = 0.376] showed that neither the correlation between the HTKS1 and the HTKS2 factors differed significantly between the two samples. In other words, children’s levels of self-regulation on the HTKS task were not significantly different in the Norwegian and U.S. samples.

### The Influence of Mother’s Education and Child Gender on Children’s Self-Regulation Across a Norwegian and a U.S. Sample

**Figure 2:**
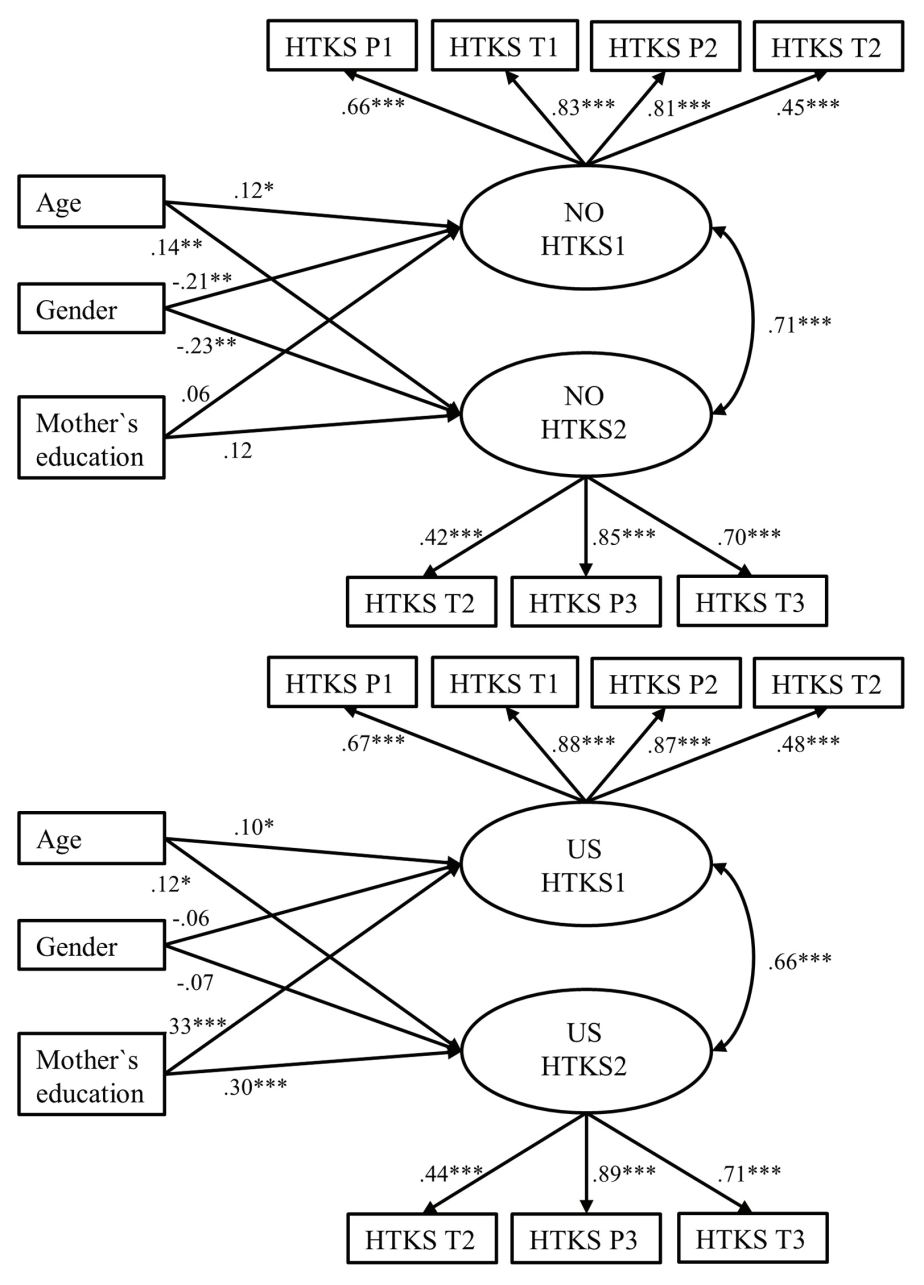
Mother’s education level and child gender predicting self-regulation using the strong MI model. Factor variances were fixed to 1, and factor means fixed to 0. The models show standardized parameter estimates. NO sample, Norwegian sample; US sample, United States sample; HTKS, Head-Toes-Knees-Shoulders task; P1, sum of practice items part 1; T1, sum of test items part 1; P2, sum of practice items part 2; T2, sum of test items part 2; P3, sum practice items part 3, and T3, sum of test items part 3. Minority status was included as a covariate, but none of the paths were significant, and they are not displayed for reasons of clarity. ^*^p < 0.05, ^**^p < 0.01, ^***^p < 0.001.

Figure 2 shows that in accordance with the correlation results, maternal education was significantly and positively related to both of the latent HTKS factors in the U.S. sample. In contrast, maternal education had a smaller, non-significant relation with the latent HTKS factors in the Norwegian sample. In other words, U.S. children whose mothers had higher education had significantly higher self-regulation as measured across all parts of the HTKS compared to children whose mothers had lower education. In contrast, maternal education was not significantly related to Norwegian children’s self-regulation. The model showed good overall fit *χ*^2^ (67) = 89.731, *p* = 0.033, RMSEA = 0.037, CFI = 0.985, TLI = 0.982, SRMR = 0.066.

To test whether the effect of maternal education on children’s self-regulation significantly differed between the two samples, we first constrained the effect of maternal education to be equal across the two samples with each of the latent HTKS factors. We compared the constrained models to the freely estimated model. The Chi-square test (Satorra Bentler corrections) showed that the effect of maternal education on HTKS1 significantly differed between the two samples [Δ*χ*^2^ (1) = 9.411, *p* = 0.002], indicating that maternal education predicted HTKS scores in the U.S. sample but not in the Norwegian. For the second factor (HTKS2), maternal education significantly predicted U.S. children’s HTKS scores but not Norwegian children’s scores. There was a trend for maternal education to predict HTKS scores differently in the two samples, but this was not statistically significant [Δ*χ*^2^ (1) = 3.482, *p* = 0.062]. Thus, the effect of maternal education on the earlier parts of the HTKS task, as represented by the first latent factor (HTKS1), was greater in the U.S. sample compared to the Norwegian sample. For the second latent factor (HTKS2), although not significant, there was a trend toward a difference in the effect of maternal education on the later parts of the HTKS task between the two samples.

Second, we constrained the effect of maternal education to be equal across the two samples on both HTKS factors in the same model. Results indicated that the effect of maternal education on the HTKS factors significantly differed in the Norwegian and U.S. samples [Δ*χ*^2^ (2) = 8.518, *p* = 0.014]. Thus, overall, maternal education influenced U.S. children’s levels of self-regulation, but not for Norwegian children, and this difference was significant between the two samples.

The results (Figure 2) showed that gender significantly predicted self-regulation (HTKS1: *β* = −0.21, *p* = 0.001 and HTKS2: *β* = −0.23, *p* = 0.001) in the Norwegian sample. In other words, girls had significantly higher scores on both of the HTKS factors compared to boys. In the U.S. sample, girls trended toward having higher self-regulation scores compared to boys, but this difference was not significant. When constraining the effect of gender on self-regulation to be equal across the two samples and comparing it to the freely estimated model, the Chi-square test (Satorra Bentler corrections) showed that there were no significant differences with the effect of gender and self-regulation between the Norwegian and U.S. samples [HTKS1, Δ*χ*^2^ (1) = 2.320, *p* = 0.128, and HTKS2, Δχ^2^ (1) = 2.514, *p* = 0.113].

## Discussion

The present study investigated cross-cultural differences in children’s self-regulation as measured by the HTKS task and in the predictors of children’s self-regulation skills. We found that children’s levels of self-regulation were similar across Norwegian and U.S. samples. Maternal education influenced children’s self-regulation significantly different across the two samples. That is, maternal education significantly predicted children’s self-regulation in the U.S. sample but not in the Norwegian sample. Furthermore, the results showed that girls had a higher level of self-regulation than boys in the Norwegian sample, but this difference was not significant in the U.S. sample. Finally, the effect of gender on children’s self-regulation did not significantly differ across the two samples.

Results supported the notion that the HTKS task measured a similar underlying construct of self-regulation across the Norwegian and U.S. samples, which strengthened and validated the comparison of the two samples (van de Schoot et al., 2012; Kline, 2016). Our results were in line with recent findings, showing that the HTKS task has shown strong psychometric properties across cultural contexts (Wanless et al., 2011a; Gestsdottir et al., 2014).

### Children’s Levels of Self-Regulation Across a Norwegian and a U.S. Sample

Results indicated that the latent factor means on the HTKS task did not significantly differ across the Norwegian and U.S. samples. Thus, the Norwegian and U.S. children represented in the present study did not have significantly different levels of self-regulation between 5 and 6 years.

The bioecological model of development emphasizes that both proximal (micro-system factors) and distal (meso-, exo-, and macro-systems factors) systems, as well as child characteristics, influence development. Characteristics of the Norwegian and U.S. cultures may support children’s self-regulation in different ways. For example, higher social and economic equality, lower child poverty, and access to ECEC and high attendance from an early age are distal factors of the Norwegian culture that influence proximal processes, which in turn might support children’s development of self-regulation. However, the Norwegian framework plan (Norwegian Ministry of Education and Research, 2011, 2017) does not mention the concept of self-regulation, which may give practitioners the impression that these skills are not important. It thus may influence practices and proximal processes that are less supportive of self-regulation. In contrast, the framework plan has a child-directed approach and emphasizes free play, child participation, and their right to choose their activities, which might be important for the development of self-regulation (Vygotsky, 1978; Center on the Developing Child at Harvard University, 2011; Engel et al., 2015). In spite of this, the Norwegian pedagogical approach may mainly benefit self-regulated children since a certain level of self-regulation is needed to engage in meaningful learning activities and play with other children without adult support (Zambrana et al., 2020).

The school readiness approach in the U.S. may also support children’s self-regulation. For example, this approach provides opportunities for children to practice self-regulation in structured and intentional ways compared to an unstructured play-based approach that is predominant in Norwegian ECECs (Gestsdottir et al., 2014). In addition, although they were of similar ages, children in the U.S. sample had made the transition to kindergarten and formal schooling, which is characterized by a more structured learning environment and a stronger emphasis on self-regulation and academic learning compared to children in the Norwegian samples who were still in a less structured ECEC setting. Thus, overall, Norway’s supportive system of families and the school readiness approach in the U.S. might have explained the non-significant differences in self-regulation.

### The Influence of Mother’s Education and Child Gender on Children’s Self-Regulation Across a Norwegian and a U.S. Sample

#### Mother’s Education Level

Based on prior research conducted in Norway and the U.S. (Wanless et al., 2011b; Backer-Grøndahl and Nærde, 2017), we expected maternal education to significantly predict children’s self-regulation in both samples, although we expected maternal education to be a significantly stronger predictor for U.S. children’s self-regulation than for Norwegian children’s self-regulation. Results partly confirmed our expectations and showed that maternal education significantly predicted children’s self-regulation in the U.S. sample but not in the Norwegian sample. Furthermore, maternal education was a significantly stronger predictor of U.S. children’s self-regulation than for Norwegian children, which is in line with prior findings showing that socioeconomic background explains a higher percentage of variation in U.S. students’ PISA performance and drop-out compared to Norwegian students’ (Lundetræ, 2011; OECD, 2016).

Maternal education significantly predicted both HTKS factors in the measure in the U.S. sample but not in the Norwegian sample. For the HTKS factors, there was a significant difference between the U.S. and Norwegian samples on the first and easiest part of the HTKS task (HTKS1 factor) and a trend toward a significant difference on the second part of the HTKS task (HTKS2 factor). Thus, the results were largely similar between the two HTKS factors suggesting that maternal education was a predictor of HTKS scores in the U.S. sample but less so in the Norwegian.

Sociodemographic factors may explain individual differences in children’s self-regulation, together with child characteristics and socialization experiences (Eisenberg et al., 2014). Examining the same measure across cultures can shed light on whether sociodemographic factors influence self-regulation differently across cultures and contexts (McClelland et al., 2010). There are differences in distal factors in Norway and the U.S. that may explain why maternal education was more important for the U.S. children’s self-regulation than for the Norwegian children. For example, it might be that the structural organization of the Norwegian society, such as a well-functioning welfare system and relatively high social and economic equality allowed Norwegian children’s development of self-regulation to be less dependent on family socioeconomic status, compared to the children growing up in the U.S. Prior evidence has shown that in rich countries, economic inequality, rather than the average income is related to children’s well-being (Wilkinson and Pickett, 2009).

The difference between the Norwegian and U.S. ECEC contexts may also be a reason for our results. Access to affordable and high-quality childcare is one way to promote healthy development in children from lower socioeconomic backgrounds and thus to reduce inequalities (Esping-Andersen, 2007; Yoshikawa et al., 2012; Hall et al., 2013). Although we did not measure ECEC quality in the present study, research has found that children in the U.S. are more likely to experience high-quality ECEC if they are from families with higher socioeconomic status (NICHD Early Child Care Research Network, 2006; Sohr-Preston et al., 2013). In contrast, Norway has universal access to state-regulated and subsidized ECEC, and most children stay fulltime in ECEC centers from age 1 year until they start formal schooling. There is also little evidence that children from families with higher socioeconomic status select differentially into better ECEC centers (Rege et al., 2018). Prior research has found that the introduction of universal ECEC in Norway had positive long-term effects on children’s educational attainment and labor market participation (Havnes and Mogstad, 2011).

#### Child Gender

In the present study, Norwegian girls had significantly higher levels of self-regulation than boys, but there were no significant gender differences in the U.S. sample. Gender differences in children’s self-regulation did not significantly differ across the two samples. Our results are in line with prior studies conducted in Norway and the U.S. reporting gender differences in favor of girls or no gender differences (McClelland et al., 2007; Matthews et al., 2009; Wanless et al., 2013; Størksen et al., 2015; Backer-Grøndahl and Nærde, 2017). Findings across other cultures are also inconsistent, which may be due to different educational approaches and assessment tools (e.g., directly assessed vs. teacher-report; Wanless et al., 2013; Gestsdottir et al., 2014; McClelland et al., 2015). For example, other cultures (e.g., France, Germany, and Asia) that have a structured learning environment and place more emphasis on academic achievement may also systematically support children’s self-regulatory skills from an early age. Thus, it may be that a structured learning environment allows both boys and girls to develop self-regulation, resulting in smaller gender differences on these skills (Wanless et al., 2013; Gestsdottir et al., 2014).

The gender differences found in the Norwegian sample might be explained by different expectations for girls’ and boys’ self-regulation (Chick et al., 2002; Meland and Kaltvedt, 2017). Different gender expectations may be overrepresented in unstructured learning environments, such as in the Norwegian ECEC system. For example, when activities are unstructured and when adults are not involved in children’s play, children spend the majority of their social interactions with members of the same gender in preschool (Fabes et al., 2003). The experiences that girls and boys get in their segregated groups differently contribute to their development, and girls’ interactions are more likely to be cooperative and less active than boys’ interactions. Girls are also more likely to select activities and engage in behaviors that are adult structured and governed by social rules. Thus, girls may have more exposure to regulated styles of play, whereas boys may have more exposure to unregulated styles of play (Fabes et al., 2003). There is also some evidence that boys can be more sensitive to environmental experiences, including chaos, that might appear in an unstructured environment (Cameron Ponitz et al., 2009b). However, it is important to note that the gender differences in children’s self-regulation in Norway were small, and gender differences did not significantly differ between the Norwegian and U.S. sample.

### Practical Implications

Prior studies have shown that self-regulation is related to school readiness and later academic achievement across cultures (Duncan et al., 2007; Wanless et al., 2013; Gestsdottir et al., 2014; Backer-Grøndahl et al., 2018), which emphasize teacher’s responsibility to facilitate the learning environment so that children receive opportunities to develop self-regulation in the early years.

There are many ways to stimulate children’s early self-regulation, and prior research has shown that social play (Center on the Developing Child at Harvard University, 2011) as well as teacher-initiated games targeting self-regulation improves children’s self-regulation (McClelland et al., 2019). However, as girls and boys are likely to select different activities when the learning environment is unstructured, the varying experiences could promote self-regulation differently in girls compared to boys. Tuastad et al. (2019) suggested combining aims and insights from the best of the two pedagogical worlds. Thus, a combination of the play-based and child-centered approach and the school readiness approach with systematic training over time may be the best way to promote gender and social equality.

In the U.S., only about half of the children attend ECEC from age 3 to 4 years, and for the youngest children, it is only 18%. High-quality ECEC can be especially beneficial for disadvantaged children (e.g., Yoshikawa et al., 2012). Thus, policy-makers can focus on ways to ensure better access to high-quality ECEC for all children at an early age in the matter to reduce inequalities among children in the U.S. (Esping-Andersen, 2005, 2006).

### Limitations and Future Directions

In the present study, the Norwegian and U.S. children were drawn from convenience samples, which only included children with prior preschool experience. Thus, the samples matched each other in several ways (Minkov, 2013). However, they may not be representative of the populations in the U.S. or Norway. Although the sample from Norway was largely representative of the typical educational experiences of children from that culture (i.e., attending an ECEC), children in the U.S. sample consisted only of children who attended at least 1 year of preschool, which is a minority (46%) of the total population (Early Care and Education Profiles, 2018). There is some evidence that attending high-quality preschool has the potential to support children’s development of cognitive and self-regulatory abilities and to combat the effects of social and economic inequalities (Hall et al., 2013).

In addition, children tested in Spanish in the U.S. sample were excluded from analyses because the current Norwegian sample did not include a similar group. ELL children in the U.S. can differ from the larger population in many ways that have important implications for children’s development (Wanless et al., 2011b; Han, 2012; McClelland and Wanless, 2012). Thus, there might be larger cultural differences than were adequately captured by the current data. Future research should investigate this topic more broadly with larger national representative samples, which would allow for the possibility to investigate more fine-grained similarities and differences across different subgroups of children in each population (Minkov, 2013).

In addition, in the present study, there was a large proportion of missing data on maternal education in the U.S. sample. Missingness on this variable was associated with a higher likelihood of minority status and lower scores on the HTKS task. Even though these auxiliary variables were included as covariates in all subsequent analyses, the significant association found between maternal education and children’s self-regulation in the U.S. sample might still be underestimated. Different patterns of missing data can influence results in the way that it can partially mask or underestimate associations between variables, which can be difficult to account for in observed variables alone (Enders, 2010). Thus, even though a significant effect was found between maternal education and self-regulation in the U.S. sample, the result might larger than what the current estimates provide.

Many studies use the country as a proxy for culture (Wanless et al., 2011a; Minkov, 2013). In the present study, we also investigated the influence of gender and mother’s education level on children’s self-regulation across two Western cultures. Vélez-Agosto et al. (2017) postulated that differences between cultures are evinced at a micro-level and that culture is not a separate system operating from a macro level but is within every action. Thus, culture manifests itself within everyday practices of social groups, such as families or classes. This has implications for research focusing on child development because it highlights the relevance of considering specific daily practices within communities or institutions, like families, ECEC centers, and schools. Global cultural influences are by no means irrelevant; however, future research would benefit from also examining the influence of other contextual factors at the micro-level across cultures (e.g., expectations on children’s self-regulation and structural and process quality in ECEC) on the development of children’s self-regulation.

According to the bioecological model of development (Bronfenbrenner, 1979; Bronfenbrenner and Morris, 2006), development occurs in an interaction between the child and the social context over time (chronosystem). Thus, future research should be conducted with samples of younger children, children not attending ECEC and school-aged children. Finally, future studies should use more than one direct measure of self-regulation, and they should also include measures that differ in terms of method of assessment (e.g., teacher-ratings) because prior research has shown that the relations between the HTKS task and teacher-rated self-regulation differ across countries (Wanless et al., 2011a). Although the present study used a behavioral self-regulation task, future studies could profit from including other aspects of self-regulation, such as emotional and more cognitive self-regulation tasks. For example, maternal education has been found to significantly predict the cognitive aspect of self-regulation [cool effortful control (EC)] but not the emotional aspect of self-regulation (hot EC) (Backer-Grøndahl and Nærde, 2017). Although cognitive and emotional aspects of self-regulation are related, examining differences in the factors that influence them is an important avenue for future research.

## Conclusion

Findings from the present study suggest that children’s levels of self-regulation, as measured by the HTKS task, were not significantly different between samples from Norway and the U.S. Furthermore, results indicate that maternal education level was related to U.S. children’s self-regulation but not to Norwegian children’s self-regulation. We also found gender differences (favoring girls) in the Norwegian sample but not in the U.S. sample, although effects were small in the Norwegian sample and the influence of gender did not significantly differ across the two samples. The present study highlights the importance of cross-cultural studies, as results from one cultural context may not be valid for other cultural contexts.

## Data Availability Statement

The data analyzed in this study is subject to the following licenses/restrictions: We are not allowed to share data outside the key personnel for the grant by the Norwegian Centre for Research Data (NSD) and the Internal Review Board (IRB) at Oregon State University. Requests to access these datasets should be directed to RL, ragnhild.lenes@uis.no.

## Ethics Statement

The studies involving human participants were reviewed and approved by Norwegian Centre for Research Data (NSD) and the Internal Review Board (IRB) at Oregon State University. Written informed consent to participate in this study was provided by the participants’ legal guardian/next of kin.

## Author Contributions

RL: conceptualization, investigation, writing, methodology, formal analysis, original draft preparation, and preparing data sets. CG: conceptualization, investigation, writing, methodology, formal analysis, preparing data sets, and supervision. IS and MM: conceptualization, investigation, writing, funding acquisition, and supervision. All authors contributed to the article and approved the submitted version.

### Conflict of Interest

The authors declare that the research was conducted in the absence of any commercial or financial relationships that could be construed as a potential conflict of interest.
